# Attention-deficit/hyperactivity disorder in India: epidemiology, diagnostic inequities, treatment gaps, and public mental health implications

**DOI:** 10.3389/fpsyt.2026.1860515

**Published:** 2026-06-16

**Authors:** Althaf Mahin, Bristow Ben Joseph, Fathimathul Sunaina, Fathimathu Rasana, Rajesh Raju

**Affiliations:** Centre for Integrative Omics Data Science (CIODS), Yenepoya (Deemed to be University), Mangalore, Karnataka, India

**Keywords:** ADHD, diagnosis, epidemiology, India, psychiatry, treatment

## Abstract

Attention-Deficit/Hyperactivity Disorder (ADHD) is a common neurodevelopmental disorder and an emerging public health concern in India. This review summarizes current evidence on the epidemiology, diagnosis, treatment, environmental influences, and public health implications of ADHD in the Indian context. Reported prevalence estimates vary widely across regions and populations, reflecting differences in study design, diagnostic practices, awareness, and access to healthcare. Although DSM-5–based diagnostic frameworks and culturally adapted assessment tools are increasingly available, access to specialist evaluation and treatment remains uneven, particularly in rural and underserved areas. Current management strategies emphasize a multimodal approach combining pharmacotherapy, behavioral interventions, educational support, and family-centered care. However, treatment access and continuity are often limited by financial constraints, inadequate healthcare infrastructure, specialist shortages, and sociocultural barriers. The review also examines the potential influence of early-life adversity, environmental exposures, lifestyle factors, and pandemic-related disruptions on ADHD risk and symptom expression. Overall, the available literature highlights persistent challenges, including underdiagnosis, regional disparities in care, and important gaps in evidence. Strengthening epidemiological surveillance, improving equitable access to services, and supporting context-specific research are essential for improving ADHD outcomes in India.

## Introduction

**Figure 1:**
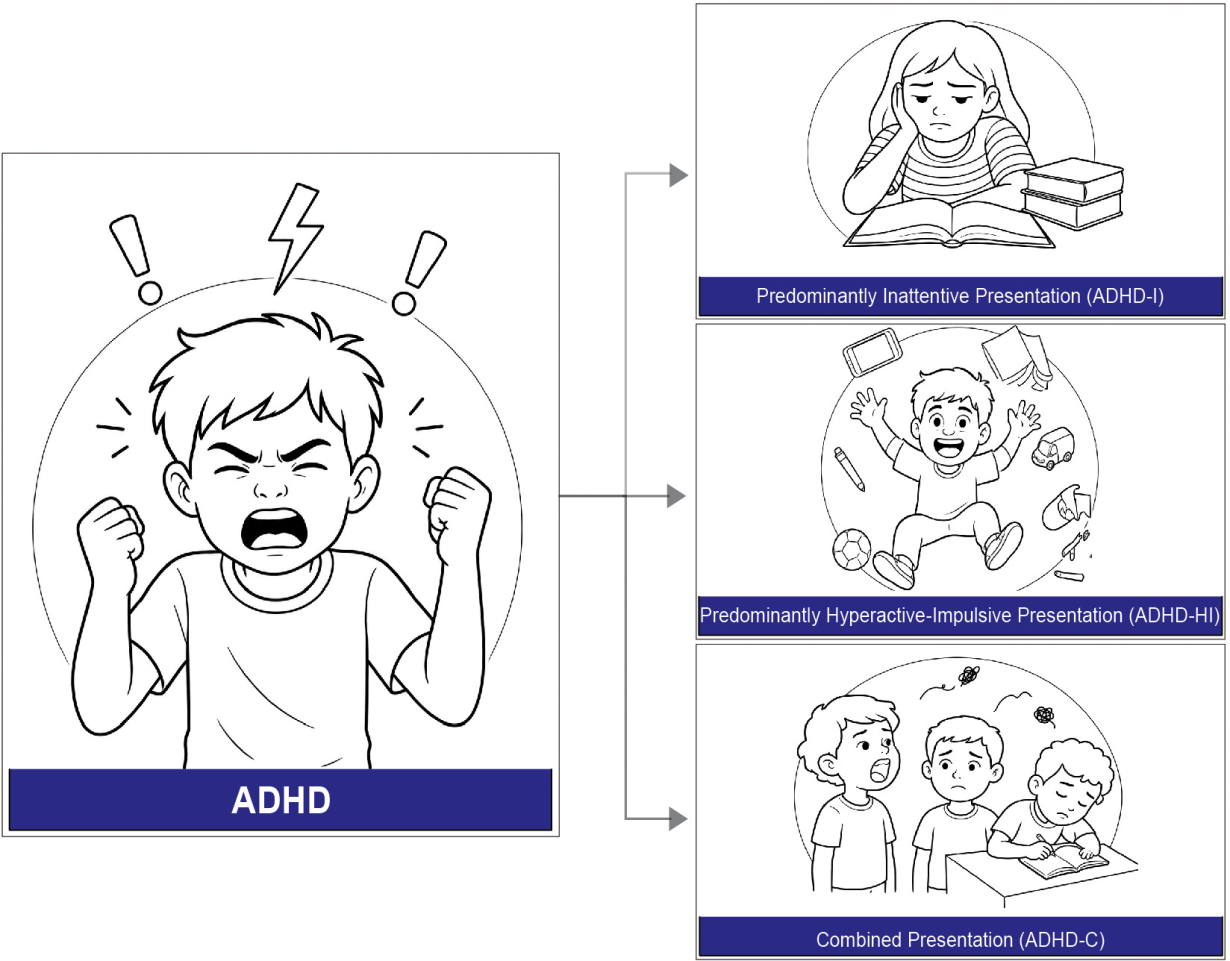
Diagrammatic representation of three main presentations of ADHD as classified by the DSM-5. Predominantly Inattentive type (ADHD-I)-Defined by distractibility and difficulty maintaining attention. Predominantly Hyperactive-Impulsive type (ADHD-HI)-Defined by extreme motor activity and impulsivity. Combined type (ADHD-C)-which show characteristics of both inattentiveness and hyperactivity-impulsivity.

Attention-Deficit/Hyperactivity Disorder (ADHD) is a common neurodevelopmental disorder that typically manifests during childhood and is characterized by persistent patterns of inattention, hyperactivity, and impulsivity (1). A systematic review and meta-analysis by Salari et al. (2023), encompassing 61 studies with 96,907 participants, estimated the global prevalence of ADHD to be 7.6% among children aged 3–12 years and 5.6% among adolescents aged 12–18 years (2). Clinically, ADHD is categorized into three presentations: Predominantly Inattentive (ADHD-I), Predominantly Hyperactive-Impulsive (ADHD-HI), and Combined presentation (ADHD-C) (**Figure 1**). The inattentive presentation (ADHD-I) is marked by distractibility, disorganization, and difficulty sustaining attention, whereas the hyperactive-impulsive presentation (ADHD-HI) is characterized by excessive motor activity, impulsivity, and restlessness. The combined presentation (ADHD-C), which integrates both inattentive and hyperactive-impulsive features, is often associated with greater functional impairment than the predominantly inattentive or hyperactive-impulsive presentations, however, the severity of impairment can vary depending on factors such as comorbid conditions, developmental stage, environmental support, timing of diagnosis, and access to treatment (3). A notable sex disparity exists in the prevalence of ADHD, with boys being approximately twice as likely as girls to receive a diagnosis (4). Furthermore, clinical evaluation and diagnosis are often complicated by high rates of psychiatric comorbidities, including Oppositional Defiant Disorder, Conduct Disorder, Anxiety Disorders, Specific Phobias, and Enuresis (5).

ADHD is increasingly understood as a neurodevelopmental condition arising from altered trajectories of brain maturation rather than from a fixed structural abnormality. Longitudinal neuroimaging studies indicate delayed cortical maturation in children with ADHD, particularly within prefrontal regions involved in attention, executive functioning, and behavioral control (6). Consistent with these findings, structural and functional neuroimaging studies have demonstrated abnormalities in fronto-striatal, fronto-parietal, and default mode networks, which are critically involved in attention regulation, inhibitory processing, reward responsiveness, and motor coordination (7–10).

At the molecular level, dysregulation of catecholaminergic neurotransmission, particularly involving dopamine (DA) and norepinephrine (NE), represents a central mechanistic framework for ADHD pathophysiology (11). Dopaminergic signaling within prefrontal and striatal circuits influences reward processing, motivation, and sustained attention, whereas noradrenergic pathways regulate arousal and executive functioning (12). Altered DA and NE signaling is therefore thought to contribute to the attentional deficits, impulsivity, and hyperactivity characteristic of ADHD (13). Further, variants in genes associated with dopaminergic and serotonergic neurotransmission, including DRD4, DAT1, DRD2, COMT, and MAOA, have also been associated with ADHD susceptibility and psychiatric comorbidities across multiple populations, including Indo-Caucasoid cohorts (14, 15). Additional evidence from epigenetic and metabolomic studies suggests that alterations in DNA methylation, oxidative stress, immune signaling, and energy metabolism may further influence ADHD phenotypes and symptom heterogeneity (16–21). Although several biological mechanisms have been implicated, their translation into clinically validated biomarkers for diagnosis, prognosis, or treatment stratification remains limited, underscoring the need for further longitudinal and population-specific studies.

## ADHD in India

In India, ADHD is markedly under-diagnosed, with pervasive gaps in awareness, delayed identification, and limited access to specialized interventions. A recent editorial by Pandey et al. emphasized the need to recognize ADHD under India’s Rights of Persons with Disabilities (RPwD) Act, 2016, to facilitate early screening, structured teacher training, and integration of ADHD services into primary mental health care, similar to European countries that have incorporated ADHD into their education and disability benefit systems (22). Complementing these concerns, recent clinical practice guidelines in India advocate individualized, developmentally appropriate interventions integrating clinical, behavioral, and educational strategies. They recommend beginning assessments with developmental screening, using culturally appropriate, non-verbal, and non-speed-based tools, and incorporating parental and caregiver reports (23). Systematic epidemiological research in India reveal varying prevalence estimates, according to sample characteristics, age group, and assessment methods (24, 25).

## Methodological approach

This narrative review aimed to synthesize the current evidence on the epidemiology, diagnostic inequities, treatment gaps, and public mental health implications of Attention-Deficit/Hyperactivity Disorder (ADHD) in India. Literature was identified through searches of PubMed and Google Scholar using combinations of keywords including “ADHD”, “attention-deficit/hyperactivity disorder”, “India”, “prevalence”, “epidemiology”, “environmental risk factors”, “early-life adversity”, “diagnosis”, and “treatment”. Additional exploratory searches using Google were conducted using related keywords to further examine specific themes discussed in the review. Priority was given to studies published in English from 2000 onward, with particular emphasis on population-based studies conducted in Indian populations. Both studies employing standardized diagnostic criteria and those utilizing validated ADHD screening or self-report instruments were considered to provide a comprehensive overview of the available evidence. Relevant review articles, clinical guidelines, and policy documents were also examined to capture additional pertinent literature. The retrieved evidence was narratively synthesized to summarize current knowledge, identify emerging themes, and highlight key research gaps relevant to the Indian context.

## Epidemiology of ADHD in India

Across India, reported ADHD prevalence demonstrates considerable regional variability, largely attributable to differences in study design, diagnostic approaches, awareness, and access to screening and mental health services. Multiple large-scale cross-sectional studies using diagnostic-support tools have been reported from the southern states of India. A study involving 3253 students across six schools in Kancheepuram district, Tamil Nadu, using the Conners’ Teacher–Parent Rating Scale, reported an ADHD prevalence of 8.8% (26). Similarly, a study conducted among 770 students from four schools in Coimbatore district, Tamil Nadu, using Conners’ Abbreviated Rating Scale (CARS), reported a prevalence of 11.32% (27). In Karnataka, a study involving 1145 students from three schools in Mysore using the Conners’ 3 Parent Short Form reported a prevalence of 14.4% (28). Likewise, a school-based study across 15 schools in Kottayam, Kerala, using the Vanderbilt Scale-IV identified an ADHD prevalence of 6% among children aged 4–5 years, with a slightly higher prevalence in boys than girls and predominance of the hyperactive–impulsive subtype (29).

Several single-center studies have also been reported from southern India. A primary school-based study from Belgaum district, Karnataka, using the SNAP-IV scale reported an ADHD prevalence of 5.76% among children aged 6–11 years (30). Another hospital-based study from Pathanamthitta, Kerala, using Conners’ Abbreviated Rating Scale reported a prevalence of 22.9% among children attending a tertiary care center (31). Additionally, a single-center study from South India involving 22 children with genetically confirmed Pyridoxine-Dependent Epilepsy (PDE) reported ADHD in 15 children (32). Self-report scales have also been employed in adolescent and adult populations. An adolescent-focused study conducted across 73 schools in Kerala using the BAARS-IV Childhood Symptoms Self-Report Scale reported an ADHD symptoms in 7.52% participants, with higher symptom burden associated with academic difficulties, psychological distress, substance use, and adverse experiences (33).

In contrast to southern India, comparatively fewer studies have been reported from northern and northeastern regions of the country. A child guidance clinic-based study conducted in a pediatric hospital in Kolkata during the early 2000s, using DSM-IV criteria, identified 37 ADHD cases among 238 referrals, with predominance of the inattentive subtype, a marked male predominance, and frequent psychiatric comorbidities (1, 34). Similarly, a study conducted among students from two schools in Cachar district, Assam, using Conners’ Abbreviated Rating Scale and the Vanderbilt Scale, reported an ADHD prevalence of 12.66%, with higher prevalence observed among boys from lower middle socioeconomic backgrounds (35). Furthermore, a study from the R.S. Pura block of Jammu and Kashmir using the Vanderbilt ADHD Diagnostic Teacher Rating Scale reported a prevalence of 6.34% (36).

Among adults, a large cross-sectional screening study conducted in Delhi using the Adult ADHD Self-Report Scale (ASRS v1.1) among 1665 individuals aged 18–25 years reported ADHD symptoms in 14% of participants (37). A subsequent study by the same group identified obesity as a significant comorbidity associated with combined-type ADHD (38). From western India, a study conducted in the Konkan region of Maharashtra using the Vanderbilt Assessment Scale-D4 reported an ADHD prevalence of 1.5% among primary school children (39). Overall, the available evidence highlights variations in ADHD prevalence and regional and methodological differences across diverse populations in India. **Table 1** summarizes the key Indian epidemiological studies highlighted in the current review.

**Table 1:** Key epidemiological cross section studies on ADHD in India.

Study	Sample characteristics	Study setting	Diagnostic tool/instrument	Prevalence
Mukhopadhyay et al., 2003 https://doi.org/10.1007/BF02723796 (1)	• N:238 • Age group: 5-12	Pediatric hospital- Kolkota	DSM IV criteria	15.5%
Jaisoorya et al., 2016 https://doi.org/10.1177/1087054716666951 (33)	• N:7150 • Age group: 12-19	Students across 73 schools in Kerala	BAARS-IV – Childhood Symptoms self-report,	7.52%
Ghosh et al., 2018 https://doi.org/10.5958/2394-2061.2018.00025.3 (35)	• N:300 • Age group: 6-11	Students from 2 schools in Cachar district, Assam	CARS (Conner’s Abbreviated Rating Scale) and Vanderbilt scale	12.66%
Joshi and Angolkar 2018 https://doi.org/10.1177/1087054718780326 (30)	• N:156 • Age group: 6-11	Primary school children from Belagavi, Karnataka	SNAP-IV (Swanson, Nolan and Pelham-IV rating scale)	5.76%
Sukanya and Vikraman 2021 (29)	• N:380 • Age group: 4-5	Preschool students from 15 schools in Kottayam district, Kerala	Vanderbit scale IV preschool version	6%
Mishra et al., 2024 https://doi.org/10.1007/s00127-024-02697-z (37)	• N:1665 • Age group: 18-25	Adults from different colleges across Delhi	ASRS V1.1	14%
Varghese et al., 2023 https://doi.org/10.7860/jcdr/2023/60328.17984 (31)	• N:157 • Age group: 6-11	Outpatient service in a tertiary care hospital in Pathanamthitta, Kerala	CARS (Conner’s Abbreviated Rating Scale).	22.9%
Gogate et al., 2024 https://doi.org/10.4103/jfmpc.jfmpc_1808_23 (39)	• N:133 • Age group: 6-12	Students from a school in Konkan, Maharashtra	Vanderbilt assessment scale – D4	1.5%
Catherine et al., 2019 https://doi.org/10.4103/psychiatry.IndianJPsychiatry_333_17 (26)	• N:3253 • Age group: 8-11	Students from six schools in Kancheepuram district, Tamil Nadu	Conners’ Teacher–Parent Rating Scale	8.8%
Venkata and Panicker, 2013 https://doi.org/10.4103/0019-5545.120544 (27)	• N:770 • Age group: 6-11	Students from four schools in Coimbatore district, Tamil Nadu	CARS (Conner’s Abbreviated Rating Scale).	11.32%
Sharma et al., 2020 https://doi.org/10.4103/jfmpc.jfmpc_587_19 (36)	• N:205 • Age group: 6-12	Students from a school in R.S Pura block of Jammu district, Jammu and Kashmir	Vanderbilt ADHD diagnostic teacher rating scale	6.34%
Manjunath et al., 2016 https://doi.org/10.9734/indj/2016/21954 (28)	• N:1145 • Age group: 6-10	Students from three schools in Mysore, Karnataka	Conner’s 3 Parent short form	14.4%

## Diagnostics of ADHD in India

In India, the diagnosis of ADHD primarily follows DSM criteria, historically DSM-IV, and currently DSM-5, while ICD-10 remains in limited clinical or administrative use, and ICD-11 adoption is gradually increasing. Culturally adapted instruments, such as the AIIMS/INDT-ADHD diagnostic tool, offer structured DSM-5–based assessments with severity scoring tailored to Indian populations (40). Commonly employed rating scales include SNAP-IV, Conners’ scales, Vanderbilt ADHD Diagnostic Rating Scale, and the Adult ADHD Self-Report Scale (ASRS) for adults. Broader behavioral checklists, such as the Child Behavior Checklist (CBCL) and the Behavior Assessment System for Children (BASC), assist in evaluating comorbidities and guiding differential diagnosis (41).

Indian guidelines emphasize multi-informant and multi-setting evaluations, often employing a two-stage process consisting of initial screening followed by a structured clinical interview, to reduce false-positive diagnoses. Diagnostic assessments are typically conducted by child and adolescent psychiatrists, developmental pediatricians, general psychiatrists, and clinical psychologists, with primary care pediatricians providing initial screening in areas lacking specialists (42). School counselors and educational psychologists primarily facilitate referrals. Specialist services are largely concentrated in metropolitan centers, whereas rural and smaller urban regions face limited access. Initiatives such as ECHO-ADHD aim to bridge these service gaps by enhancing training and tele-mentoring (43, 44).

Comorbidity patterns observed in Indian clinical samples mirror global trends. Externalizing disorders, including Oppositional Defiant Disorder (ODD) and Conduct Disorder (CD), occur in 20–50% of cases, while internalizing disorders such as anxiety and depression affect substantial minorities. Learning disorders (specific learning disorders, SLD) co-occur in 20–40% of cases, and conditions such as autism spectrum disorder (ASD) or intellectual disability may present with overlapping symptoms, necessitating careful differential assessment (45–47).

However gender disparity in ADHD diagnosis remains a concern among. A study analyzing gender differences in adult ADHD among 101 treatment-seeking adults in India (78 men, 23 women) reported that females with ADHD are frequently under-recognized or misdiagnosed, as their symptomatology predominantly involves internalizing features such as affective disturbances, low self-esteem, and pronounced inattention or hyper-focus, in contrast to the prototypical externalizing hyperactivity more commonly observed in males (48). Reflecting the current gaps in recognition, contextually, a recent Indian news report described a woman who discovered in her thirties that she had ADHD during pre-marital counseling, after years of bullying, poor academic performance, and low self-esteem (49).

## Treatment strategies of ADHD in India

**Figure 2:**
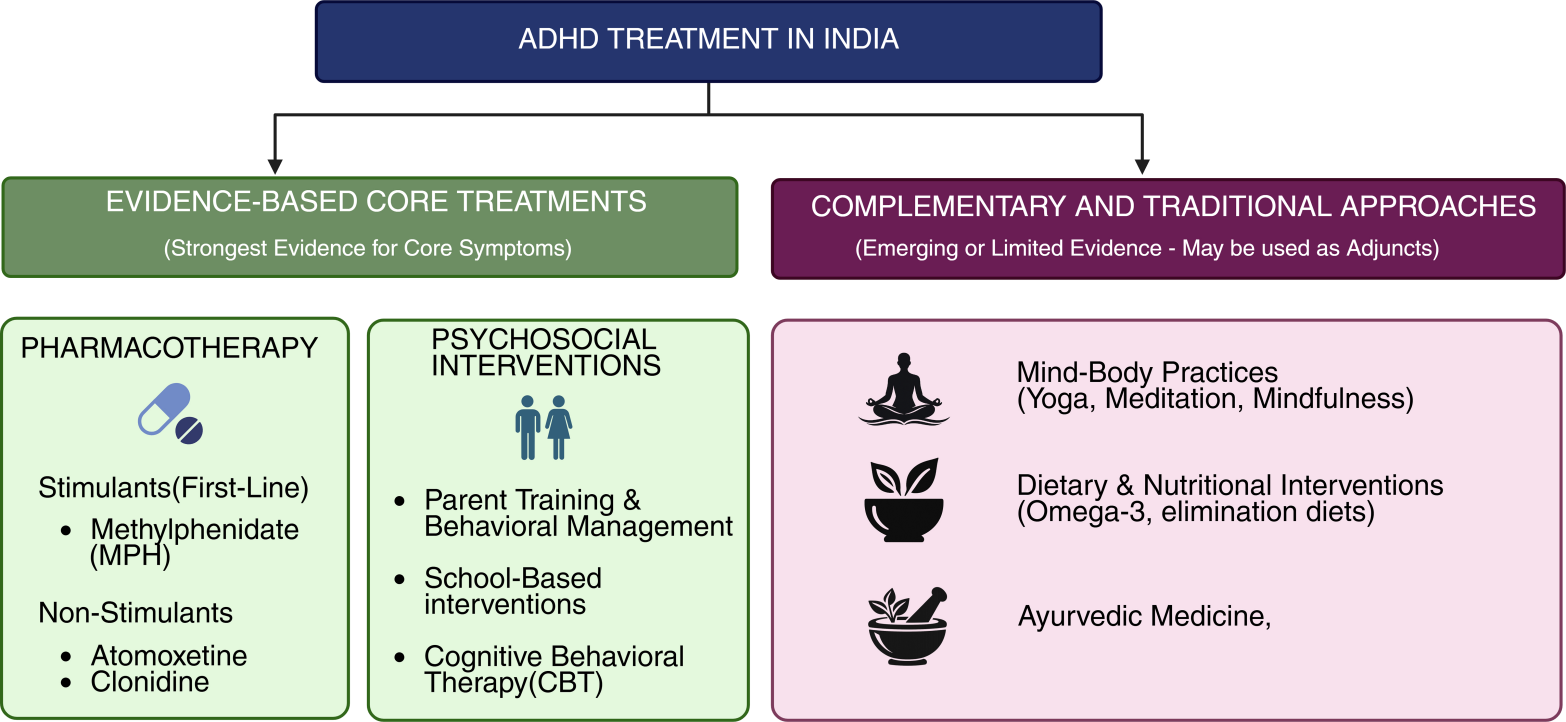
Overview of ADHD treatment approaches and management strategies in the Indian population.

Current Indian clinical practice guidelines recommend a multimodal and individualized approach to ADHD management, combining pharmacological treatment with psychosocial and educational interventions according to symptom severity, functional impairment, age, and the presence of comorbidities (41). In India, however, ADHD care remains largely concentrated within urban tertiary-care centers and specialist psychiatric services, resulting in inequitable access to diagnosis and treatment. Pharmacotherapy remains the cornerstone of ADHD management, with methylphenidate (MPH) serving as the primary medication and being available in both immediate- and extended-release formulations. Non-stimulant alternatives, including atomoxetine (ATX) and clonidine, are prescribed when stimulants are contraindicated, poorly tolerated, or when comorbid conditions such as anxiety or tic disorders are present. Immediate-release clonidine is also used to manage sleep disturbances associated with ADHD (50, 51). Despite the availability of these medications, treatment access and continuity remain challenged by financial constraints, regional disparities in healthcare infrastructure, regulatory requirements, limited availability of trained specialists in rural and underserved areas, and factors influencing adherence, including concerns about adverse effects, perceived medication dependence, stigma surrounding psychiatric disorders, and limited awareness of ADHD among caregivers and educators (52) (**Figure 2**).

Psychosocial interventions are routinely combined with pharmacotherapy to enhance outcomes. Parent-training and behavior-management programs, adapted for Indian families, have been shown to reduce core ADHD symptoms and alleviate parental stress (53). School-based interventions, including teacher education, classroom modifications, and peer-mediated strategies, contribute to improvements in both academic performance and behavioral regulation (54). However, the availability of behavioral therapy, parent-management training, psychoeducational services, and structured school-based support remains inconsistent across the country. Cognitive Behavioral Therapy (CBT) is increasingly applied for adolescents and adults, targeting residual executive deficits and co-occurring mood or anxiety symptoms (55). Complementary and traditional approaches, including Ayurvedic interventions, yoga, and meditation, are also utilized by some families as adjunctive management strategies. While few studies have reported improvements in attention, behavioral regulation, stress reduction, and overall well-being, the quality of the evidence remains limited as are frequently characterized by small sample sizes, short follow-up periods, methodological heterogeneity, inadequate control groups, and variability in intervention protocols (56, 57).

## Early-life adversity and the persistence of ADHD in India

Early-life adversity (ELA) encompasses stressful and traumatic experiences occurring during critical developmental periods, including physical and emotional abuse, neglect, malnutrition, household dysfunction, exposure to violence, and adverse caregiving environments (58, 59). Increasing evidence suggests that ELA may contribute to both the development and persistence of ADHD symptoms by disrupting the maturation of neural, endocrine, and immune systems during sensitive stages of brain development (60). The relevance of ELA to ADHD is particularly important in India, where a large young population continues to experience substantial socioeconomic disadvantages (61). Rapid yet inadequate globalization and urbanization have transformed educational, social, and occupational environments, exposing young individuals to increasing academic pressures and psychosocial stressors (62, 63). Such exposures may influence neurodevelopment during sensitive developmental periods. Supporting this, neuroimaging study conducted among infants and young children from rural Uttar Pradesh demonstrated that lower maternal education and household income were associated with weaker neural activity and reduced distractor suppression within frontal cortical regions involved in working memory (64).

Beyond neurodevelopmental effects, ELA has been associated with emotional dysregulation, psychological distress, anger, low self-esteem, helplessness, and suicidal ideation among Indian young adults (65, 66). These findings are particularly relevant because anxiety and depressive disorders, the most common psychiatric comorbidities of adult ADHD, are consistently associated with childhood adversity, with individuals exposed to one or more adverse experiences showing a substantially greater risk of anxiety and a higher prevalence of adult ADHD (67). Direct evidence from India further supports this association, as a recent study among college-going young adults in Delhi reported that individuals exposed to adverse childhood experiences were approximately 1.8 times more likely to exhibit ADHD symptoms than their unexposed counterparts (68). The long-term impact of these developmental vulnerabilities is reflected in a large study involving 5,145 college students from Kerala, where individuals reporting clinically significant childhood ADHD symptoms demonstrated higher odds of poor academic achievement, substance use, psychological distress, suicidal behaviors, and experiences of abuse (69).

## Impact of the COVID-19 lockdown on ADHD in India

The COVID-19 pandemic substantially influenced ADHD symptomatology, recognition, and care in India. School closures, disrupted daily routines, increased screen exposure, sleep disturbances, and elevated parental stress were associated with worsening hyperactivity, inattention, and conduct problems, affecting both children with pre-existing ADHD and those with subthreshold symptoms (70). Pandemic-related disruptions also interfered with adherence to pharmacological and behavioral interventions, while the expansion of telemedicine and heightened parental vigilance altered patterns of help-seeking and treatment, including among adults with ADHD (71). Mechanistically, symptom exacerbation during the pandemic can be attributed to multiple environmental and lifestyle changes: loss of structured school environments, reduced external scaffolding for attention and behavior regulation, poorly timed and excessive screen exposure impacting sleep and arousal, decreased physical activity, elevated familial stress, and limited access to school-based or in-person behavioral interventions (72). These compounded stressors amplified underlying neurodevelopmental vulnerabilities, disproportionately affecting children with ADHD or elevated developmental risk.

## Environmental and lifestyle parameters influencing ADHD susceptibility

Prenatal, perinatal, and early postnatal factors have been associated with altered vulnerability to ADHD. Children conceived via assisted reproductive technologies or exposed to gestational diabetes, particularly in socioeconomically disadvantaged populations, have shown higher rates of ADHD-related outcomes in observational studies (73, 74). Preterm birth and lower gestational age have similarly been associated with the development of inattentive symptoms, particularly among girls (75). Maternal stress, substance exposure, abnormal birth weight, and metabolic or hemodynamic disturbances during pregnancy have also been linked with increased likelihood of ADHD symptoms (76, 77). Additional perinatal factors reported in the literature include maternal smoking and prenatal exposure to mercury, including through fish consumption during pregnancy (78–80). Environmental toxicants such as organophosphate pesticides, polychlorinated biphenyls, and endocrine-disrupting chemicals including antiandrogenic phthalates have likewise been investigated for possible associations with ADHD risk (81, 82). Psychosocial adversities, including physical or sexual abuse and parental neglect, have been associated with increased impulsivity and attentional difficulties (83, 84). Furthermore, the quality of parent–child relationships, particularly the mother–daughter dyad, may influence ADHD symptom expression and broader mental health outcomes (85). Lifestyle-related parameters including excessive screen time, sleep deprivation, poor nutrition, deficiencies in iron, omega-3 fatty acids and other micronutrients, sedentary behavior, and obesity have also been correlated with ADHD symptom burden in children and adolescents (86–89).

Many of these environmental and lifestyle parameters are prevalent across India’s heterogeneous population. Heavy metal exposure, particularly lead exposure, remains an important public health concern. Case–control studies from Korea and Iran have identified elevated blood lead concentrations as being associated with ADHD in children (90, 91). In this context, reports from India are noteworthy, as elevated blood lead levels and high groundwater lead concentrations have been reported among children in regions such as Punjab and Chandigarh (92). Similarly, systematic reviews have reported significant associations between exposure to fine particulate air pollutants and increased ADHD risk (93, 94). In relation to this, recent reports describing elevated air pollutant levels in Delhi and across the Indo-Gangetic Plain warrant further attention in the context of ADHD susceptibility and neurodevelopmental health (95, 96).

## An alternate perspective on the rise of ADHD in India

Global trends suggest that populations transitioning from agrarian or manual labor–based lifestyles toward industrialized and sedentary economies often experience increasing prevalence of several chronic non-communicable and immune-mediated disorders (97–99). In this context, the observed rise in ADHD prevalence may partly reflect the influence of environmental and lifestyle transitions, in addition to improved recognition and expanding diagnostic practices. For children, the consequences of rapid urbanization in India including urban heat islands, deteriorating air quality, increased consumption of ultra-processed foods, persistent environmental pollutants, chronic psychosocial stress, reduced access to natural environments and outdoor play spaces, increasing academic pressures, and rising screen exposure may collectively influence cognitive, behavioral, immune and socio-emotional development (93, 100–103). Concurrently, reduced exposure to natural microbial environments (the farm effect) and increasingly sedentary lifestyles have been hypothesized to contribute to immune–brain dysregulation, obesity, insulin resistance, and systemic inflammation, factors that may influence neurodevelopmental and behavioral outcomes (104–108). Countries such as India, currently undergoing rapid modernization and urban expansion, are increasingly exposed to similar environmental and lifestyle pressures. While direct causal relationships remain to be fully established, these transitions warrant consideration as potentially relevant contextual factors in ADHD epidemiology alongside established genetic and neurodevelopmental mechanisms (109–111).

## Health determinants, risk factors and barriers of ADHD

**Figure 3:**
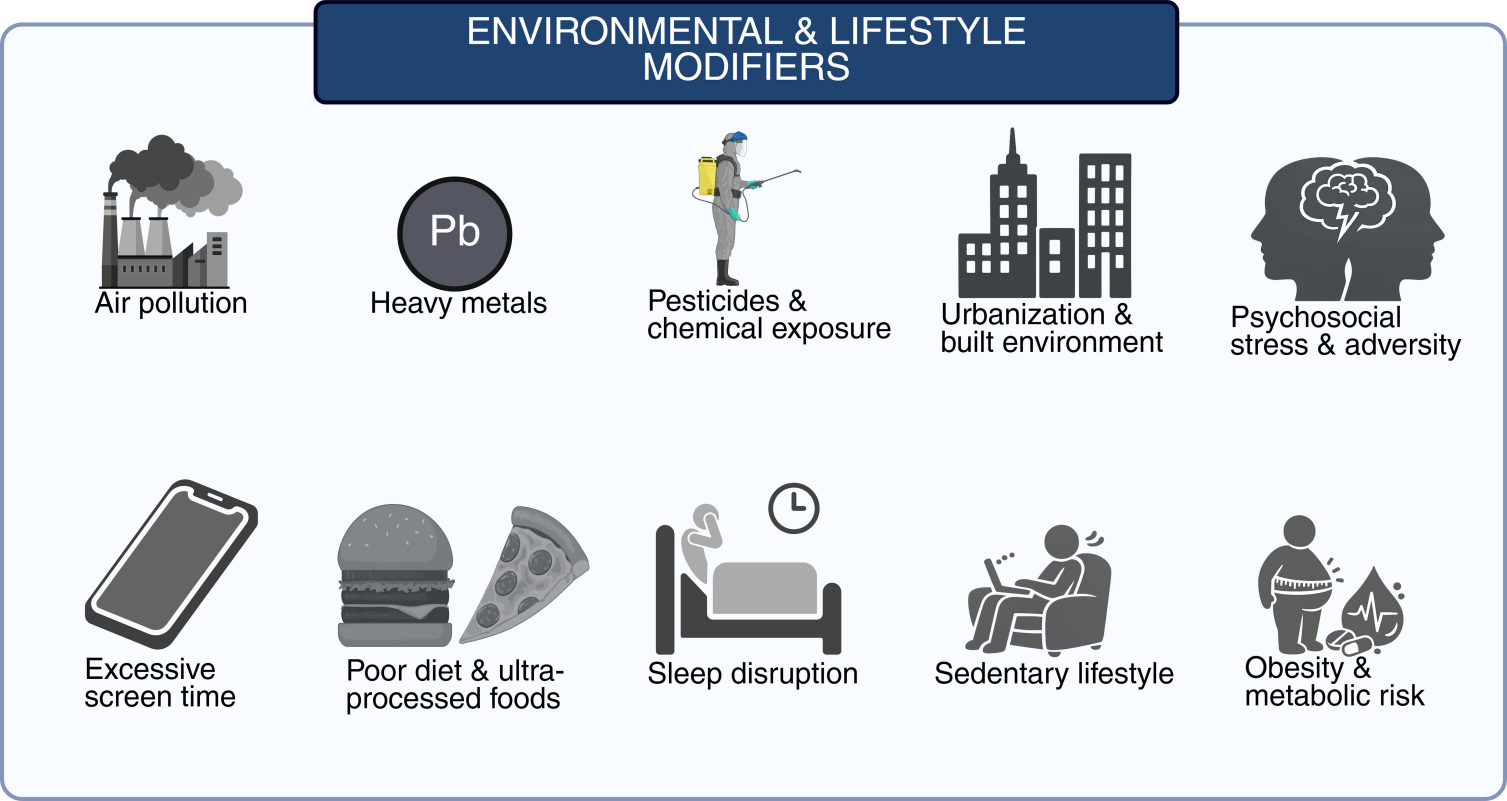
Environmental, psychosocial, and lifestyle factors associated with ADHD susceptibility.

Addressing the challenges associated with ADHD remains complex due to multifactorial barriers spanning clinical, social, and research domains (**Figure 3**). Access to care is often limited, particularly in rural and low-income populations, where shortages of trained professionals and the high cost of medications and therapy hinder optimal management (112, 113). Persistent stigma and public misconceptions regarding ADHD as a behavioral or disciplinary issue further discourage affected individuals from seeking timely diagnosis and treatment. Adult ADHD remains notably underdiagnosed, as diagnostic frameworks and interventions have traditionally focused on children, leaving a gap in adult-specific treatment models (114). Treatment adherence poses another major challenge, especially among adolescents and young adults who often resist medication or fail to maintain consistent follow-up (115). High rates of comorbidities, including anxiety, depression, and substance use disorders, complicate both diagnosis and treatment, emphasizing the need for integrated, interdisciplinary care models (5). Moreover, diagnostic subjectivity persists due to the reliance on behavioral assessments, with objective biomarkers such as neuroimaging, EEG, and genetic indicators remaining largely investigational and not currently recommended for routine clinical diagnosis of ADHD (116). Females with ADHD remain particularly overlooked, as their symptoms often present through inattentiveness and internalizing behaviors rather than overt hyperactivity (48).

The enigmatic nature of ADHD recognition in India, particularly within rural demographics, may also stem from the deep entanglement of cultural norms, familial expectations, and social constructs that shape behavioral interpretation (36, 117). In rural communities where cultural conformity exerts strong influence, children are socialized to obey authority and adhere to collective norms, leading behaviors such as impulsivity or inattentiveness to be perceived as signs of indiscipline or poor upbringing rather than as manifestations of a neurodevelopmental condition (118). The Indian education system’s strong emphasis on academic excellence further obscures ADHD-related symptoms, as affected children in rural settings are often labeled unmotivated or careless instead of being clinically assessed (119–121). Widespread stigma surrounding mental health, coupled with limited awareness among educators and healthcare providers working in rural regions, further delays recognition and intervention (122, 123). Moreover, traditional beliefs prevalent across many regions attribute behavioral difficulties to spiritual or moral causes, making clinical diagnosis particularly elusive within India’s highly heterogeneous population (124–126).

## Research priorities, future directions, and limitations

**Figure 4:**
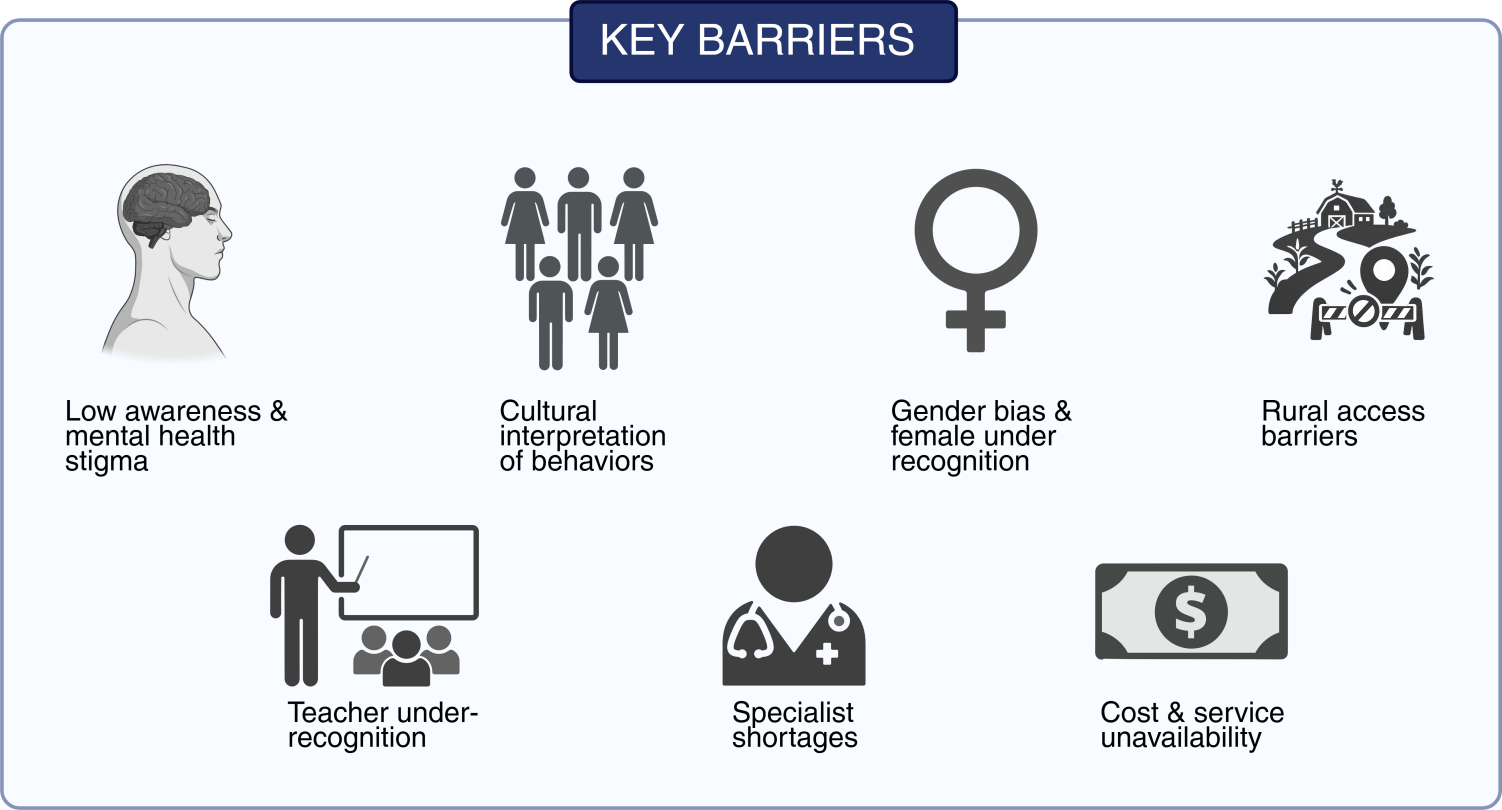
Key barriers affecting ADHD recognition, diagnosis, and treatment access.

Future ADHD research in India should focus on developing context-specific models of identification, diagnosis, and long-term care. There is a pressing need for large longitudinal cohort studies that track neurodevelopmental trajectories from childhood into adulthood, enabling a better understanding of symptom persistence, functional outcomes, psychiatric comorbidities, and sociocultural determinants of ADHD across the lifespan (**Figure 4**). Particular attention should be directed toward female ADHD, which remain substantially underrecognized despite growing evidence of diagnostic disparities. Further, studies examining the integration of ADHD services into primary healthcare, community mental health programs, and rural service-delivery models are essential to address the significant treatment gap arising from specialist shortages and inequitable access to care in underserved regions. Finally, implementation research should evaluate the effectiveness, scalability, cost-effectiveness, and policy integration of ADHD interventions, including telepsychiatry-enabled care, family education programs, teacher training initiatives, and disability-support frameworks, to ensure that evidence-based ADHD services can be sustainably translated into real-world practice across India.

Limitations of the current evidence base should also be acknowledged. Although environmental, lifestyle, and psychosocial factors such as air pollution, urbanization, dietary transitions, screen exposure, early-life adversity, and chronic stress have been implicated in ADHD pathogenesis, much of the direct evidence supporting these associations has been generated from studies conducted outside India. Evidence from India is currently derived predominantly from contextual and observational studies that establish the presence and plausibility of these exposures within Indian settings, rather than from large-scale longitudinal cohorts demonstrating causal associations with ADHD. Consequently, the applicability of these findings to the diverse sociocultural, environmental, and healthcare contexts of India remains uncertain. Furthermore, the current literature is also characterized by regional heterogeneity, limited representation of rural populations, underrepresentation of females and adults with ADHD, and variability in diagnostic methodologies, all of which constrain the generalizability of existing findings and highlight the need for context-specific research.
